# Influence of Nanoparticles on Thermal and Electrical Conductivity of Composites

**DOI:** 10.3390/polym12040742

**Published:** 2020-03-27

**Authors:** Divan Coetzee, Mohanapriya Venkataraman, Jiri Militky, Michal Petru

**Affiliations:** 1 Department of Material Engineering, Faculty of Textile Engineering, Technical University of Liberec, 461 17 Liberec, Czech Republic; divan.coetzee@tul.cz (D.C.); jiri.militky@tul.cz (J.M.); 2 Institute for Nanomaterials, Advanced Technologies and Innovation, Department of Machinery Construction, Technical University of Liberec, 461 17 Liberec, Czech Republic; michal.petru@tul.cz

**Keywords:** nanoparticles, Seebeck effect, thermal conductivity, electrical conductivity, metal nanoparticles, carbon nanoparticles, composite recycling

## Abstract

This review analyzes thermal and electrically conductive properties of composites and how they can be influenced by the addition of special nanoparticles. Composite functional characteristics—such as thermal and electrical conductivity, phase changes, dimensional stability, magnetization, and modulus increase—are tuned by selecting suitable nanoparticle filler material. The conductivity of composites can be related to the formation of conductive pathways as nanofiller materials form connections in the bulk of a composite matrix. With increasing use of nanomaterial containing composites and relatively little understanding of the toxicological effects thereof, adequate disposal and recyclability have become an increasing environmental concern.

## 1. Introduction

Nanoparticles could be either organic or inorganic in nature with dimensions below 100 nm. Nanostructured materials are receiving increasing attention for their advantageous properties such as extremely huge relative surface area and surface reactivity, no contact heat transfer problems, and availability for natural convection to enhance heat transfer during melting [1]. Nanostructured materials for this purpose are typically used in the form of nanoparticles, nanowires, nanotubes, nanofibers, etc. [2]. Composites combine the properties of a bulk material with that of a size-dependent particle [3]. Composites can be classified as either ceramic matrix nanocomposites (CMNC), metal matrix nanocomposites (MMNC), or polymer matrix nanocomposites (PMNC) to which nanoparticles can be added to either enhance, change, or add to the properties of the matrix material. Composite formation techniques include ex situ, chemical, and physical in situ methods which lead to the final composite or the formation of hybrid nanoparticles. Ex situ processes are where nanoparticles are synthesized and introduced into the polymer matrix in solution. This process has a typical drawback of nanoparticle agglomeration which can be difficult to overcome and resolve. Chemical in situ methods use a liquid environment to generate the nanoparticles as either hybrid particles or directly into the polymer matrix. This method typically ensures a more homogeneous dispersion of the nanoparticles compared to the ex situ process. Physical in situ methods are mainly gas-phase methods used to produced encapsulated nanoparticles which appears as hybrid nanoparticles. Energy is applied to a precursor gas of chemical compounds which then forms inorganic nanoparticles. This is followed by a subsequent coating step in which organic compounds are grafted onto the formed nanoparticle surfaces for coating, encapsulation, or surface functionalization [4].

Most of the properties associated with nanoparticles can be attributed to particle size [5]. Nanoparticle size has a direct impact on the electrical and thermal conductivity, polymer phase behavior and stability, mechanical properties, flame retardancy, density, magnetic, optic, or dielectric properties of the polymer matrix. This definition restricts true nanoparticles to particles with dimensions in the range of 10–20 nm [6]. Theory states that with a decrease in particle size the ratio of particle surface area respective to its volume increases hyperbolically. As the size of nanoparticles decrease, the effect of cohesive forces attracting the particles decrease hyperbolically except for Ag nanoparticles embedded in a Ni containing bulk material when the inverse effect occurs. This is due to a high attractive force between silver and nickel relative to the low surface energy of silver nanoparticles [7]. Embedding ZnO nanoparticles into a PANI (polyaniline) polymer matrix resulted in a spectral red shift whilst reducing the optical band gap energy by 1.9% [8]. Hanemann and Szabó noted that the refractive index of PbS nanoparticles with sizes below 25 nm was significantly less compared to larger particles. For SnO_2_ nanoparticles, a significant increase in energy band gap was observed with a decrease in particle size. For a ZrO_2_ nanoparticle embedded matrix a decrease in particle size resulted in a decrease in transition temperatures which is a typical effect experienced when incorporating ceramics into a polymer matrix. A decrease in particle size for TiO_2_ resulted in an increase in electrical storage capacity with similar effects observed for rutile particles. A decrease in ferroelectric to paraelectric phase transition in PbZrO_3_ at particle sizes below 100 nm was observed, as well as a decrease in dielectric constant. Below a particle size of 70 nm, the paraelectric phase was stable; whilst above 100 nm, the tetragonal ferroelectric phase was stable. This was because of pseudo-tetragonal distortion of the crystal lattice [4]. Particle size has a direct influence on the surface and interfacial properties of nanoparticles. Smaller particle diameters exhibit an increasing probability towards particle agglomeration due to electrostatic, steric, and Van der Waals forces [9]. Uniform particle dispersion is therefore essential in obtaining the desired composite properties. Methods of nanoparticle dispersion include melt-state sheer, ultrasonication, and solid-state pulverization. Fragile nanoparticles, such as carbon nanotubes, typically undergo chemical dispersion such as with the use of surfactants [10]. Inadequate dispersion could lead to greater electrical resistance as well as mechanical failure. Nanoparticles dispersion can be classified under two main categories such as physical or chemical dispersion. Physical dispersion techniques include ultrasonic agitation which uses ultrasonic waves to vibrate the particles to move them away from each other. This causes nanoparticle dispersion in the matrix whilst allowing agglomerated particles to split, which is a typical problem when using electrically conductive particles or particles with magnetic properties as they have a high attraction tendency. This method produces heat due to the vibration of particles and it therefore recommended to be performed in a thermal bath. Ultrasonication has proven to be unsuitable for dispersion of carbon nanotubes as they experience damage from the vibrations [11]. Sheer mixing is preferred for viscous polymer matrices; however, this is typically performed using a magnetic stirrer and is therefore impractical for magnetically active metallic nanoparticles. Ball milling involves metal or ceramic balls moving around in a rotary cylinder to break clusters of particles [12]. In the wet state, this process usually more efficient [13]. This method is preferred for dispersion of carbon nanotubes for their high clustering rate, for example. However, improvements in this technique have been made by using micro sized balls to disperse nanoparticle agglomerates. Chemical dispersion methods include covalent and non-covalent techniques. Covalent techniques involve functionalization of the nanoparticles with hydroxyl and carboxyl functional groups which mainly work with a repulsion mechanism, however further research is investigating this method. Non-covalent methods are most commonly used with surfactants to avoid nanoparticle aggregation [11].

Thermal interface materials (TIMs) can be classified as thermal greases, elastomeric thermal pads, solders, and phase change materials. Thermal greases are comprised of ceramic nanoparticles such as alumina and mesoporous silica which have high thermal conductivities and are electronically insulative materials. TIM matrix components are thermally stable polymers such as silica and paraffin in which nanoparticles form thermally conductive pathways. Heat is conducted through the formed pathways to be dissipated in a heat sink. Synergic effects of TIMs rely on the thermal conductivity of the nanoparticles for effective heat transfer. PCMs utilize this enhanced thermal conductability to promote a phase change for energy storage [14].

Synergic effects of nanofiller materials for use in thermoelectric composites are dependent on the influence of the filler on the Seebeck coefficient of a composite. This effect is greatly influenced by incorporating alloy or metal oxide nanoparticles. The effect is observed when a temperature difference drives a charge carrier which diffuses from the higher to the lower temperature end of the composite. This flow of charge results in a current. The opposite is observed with the Peltier effect when heating or cooling occurs at an electrified junction of two different conductors. Heat is absorbed at an upper junction and rejected at a lower junction when a current flows through a circuit [15]. Carbon nanoparticles exhibit a typical problem of agglomeration in a matrix. When this is overcome by adequate dispersion techniques or when adequately applied to a surface, the electrical conductivity of the composite can be increased significantly, depending on the purpose. For example, for use in lightning strike protection devices where the nanoparticles offer a safe conductive path on the exterior skin of a composite panel when applied by surface application such as spraying [16]. Electromagnetic interference (EMI) shielding materials must both reduce undesirable emissions and protect the component from stray external signals. EM radiation must be reflected using charge carriers which interacts directly with the electromagnetic fields. For this, EMI materials need to be electrically conductive. Secondly, EM radiation should be absorbed. This is facilitated by electric and/or magnetic dipoles of the filler material interacting with the radiation. Thus, the reflectivity and absorption characteristics of the EMI device are determined by the electrical conductivity of the composite which is enhanced by the nanoparticle filler materials [17]. Ferromagnetic materials, such as iron, use magnetic dipole characteristics to absorb EM radiation. Combining this with an electrically conductive filler material such as carbon nanotubes to form an interconnected network could further increase the EMI shielding [18,19]. Thirdly, the internal reflections such as grain boundary scattering also enhances the scattering and absorption of EM waves [17]. This internal scattering effect was observed for Bi_2_Te_3_ nanoparticles. Nanoparticles such as carbon nanotubes and graphene oxide exhibit some of the highest electrical conductivities for use EMI applications. The conductive pathways formed by the nanoparticles absorb the EM radiation and dissipate the resulting stress throughout the matrix [20]. Carbon nanotubes are excellent for providing linear contacts; however, the synergic effect is increased when combining with 1-D nanostructures such as carbon black of graphene oxide nanoparticles [21]. PEDOT:PSS is a core–shell structured conductive polymer with a high degree of mechanical flexibility and excellent dispersion in water [22]. Synergic effects of PEDOT arise from the formation of electrical pathways by allowing delocalized π-electrons to move freely in the unsaturated backbone of the polymer [23]. The PSS dopant creates a delocalized charge which is responsible for transferring of electrons from the PEDOT polymer chains which leads to the formation of polarons/bipolarons that allow charges to pass through the polymer backbone [22]. 

Methods such as friction stir processing (FSP) are used to distribute and incorporate nanoparticles onto the surface of solid matrixes [24]. In FSP, localized heating occurs at the point of friction contact which softens and plasticizes the matrix materials. By the movement of the pin, a volume of processed material is produced by the materials grinding from the front to the back of the pin. This causes the applied material to undergo intense plastic deformation and results in significant grain refinement. FSP is a relatively new technique with potential to generate a microstructure which is amenable to a high strain rate super plasticity [25]. Regarding the polymer–nanoparticle interface, when the two components experience attractive forces this will result in an increase of the glass transition temperature of the polymer matrix due to restricted mobility of the polymer chains. An increase in particle size at lower weight percentages up to 5% of Al_2_O_3_ and SiO_2_ nanoparticles in a PEEK (polyetheretherketone) matrix resulted in an increase in tensile and compressive strength whilst flexural strength was unaffected. With a decrease in nanoparticle size, the degree of filling in the overall polymer will decrease correspondingly [5]. The effect of particle size on the various matrix properties are presented in Table 1.

Nanoparticles could improve the mechanical properties of bulk materials due to higher particle surface binding without loss of composite ductility when used in similar volume fractions compared to micro sized fillers. Properties of composites using nanoparticles are mainly as result of the small nanoparticle size; however, the effective reinforcement and conductive contribution of nanoparticles to a matrix is largely dependent on the distance from one particle to another in the polymer matrix. Smaller distances between particles in a matrix would result in an improved contribution to the reinforcement of a composite and formation of conductive pathways [26]. The conductive pathways can be improved by functionalizing the nanoparticles to create molecular networks in the matrix. In the formed network, the nanoparticles act as electronic contacts between the molecules and exhibit photoconductive properties [27]. Nanoparticle composites used in industry proved to exhibit enhanced properties such as higher modulus, dimensional stability, heat distortion temperature, toughness, and rheological properties, as well as improved scratch ad mar resistance [28]. Nanomaterials are still considered to be a relatively new field of research and nanoparticle toxicology is still not fully understood [29]. EMI materials are used in applications where electromagnetic radiation must be dissipated to prevent it from passing a specific boundary. This is done either via reflective or absorptive mechanisms depending on the materials used. Electromagnetic radiation increases with an increase in frequency. Metals such as copper, gold, silver, and aluminum are good for EM reflective materials whereas carbon, super-permalloy, and mumetal are effective EM radiation absorbers. These materials can be incorporated into a polymer matrix in the form of nanomaterials, with effectiveness increasing at higher volume fractions. It was noted that spherical structures posed lower effectiveness and use of the filler material in flat/flake form yielded higher effectiveness at lower volume fractions [30]. Silver nanoparticles (AgNPs) which were self-assembled into multi-layered films were utilized to enhance the optically stimulated luminescence emission and increase the sensitivity of radiation detectors. Similarly gold nanoparticles were used for intravascular imaging by photoacoustic measurements and detection of inorganic, organic, or biological analysis [31].

Metallic nanoparticles, such as AgNPs, exhibit antimicrobial properties in addition to adding thermal or electrical conductivity to a matrix. This has led to the development of composites for wound dressing applications [32]. Nanoparticles are ideal for drug delivery because of their small size which enables them to penetrate cells and increase drug concentration at the target site. Applications of these include the use of nanoparticle embedded hydrogels which have excellent biocompatibility, modifiable porosity, a flexible and feasible platform for use in targeted drug delivery systems. Hydrogels for encapsulation of nanoparticles called nanocapsules are also classified as nanoparticles with enhanced benefits for drug delivery [33]. For this purpose, cyclodextrins have been studied as a matrix for use as an encapsulation material due to their ability to incorporate or absorb molecules into their central cavity [34]. 3D printing in the consumer market has grown by 346% over the last decade. Printing technology has evolved into printers that operate on resolutions in tens of nanometers. PEDOT is a highly conductive polymer which can be 3D printed and used in a wide range of applications ranging from biosensors, to energy storage [35]. For application in the field of medicine, PEDOT has unique characteristics in terms of biocompatibility and interfacing with living cells when determining hyperosmotic stresses induced by imbalances in cellular fluid osmolarity [36]. Fatigue inspection of materials is common practice in industry and is often a costly and labor-intensive procedure. Studies have proven that by incorporating electrically conductive nanoparticles into a matrix could improve failure detection in materials. Recyclability of composite materials is important given their polymeric nature. Projections indicated that by 2030 obsolete computer waste would reach 1 billion tons. Up to 85% of electronics waste ends up in landfills, of which many components are toxic [37].

## 2. Nanoparticles for Improving Thermal Conductivity

Phase change materials (PCMs) have been used extensively due to their capacity to store and release large amounts of thermal energy by promoting a phase change from a solid to liquid phase at a specific temperature. The thermal conductivity (TC) of a material influences the melting-solidification rate which is also referred to as the charging and discharging rate [38]. The addition of nanoparticles could increase or decrease the thermal conductivity of a material or liquid by suppressing natural convection [39].

### 2.1. Hydrogel PCM Nanoparticle Systems

**Figure 1:**
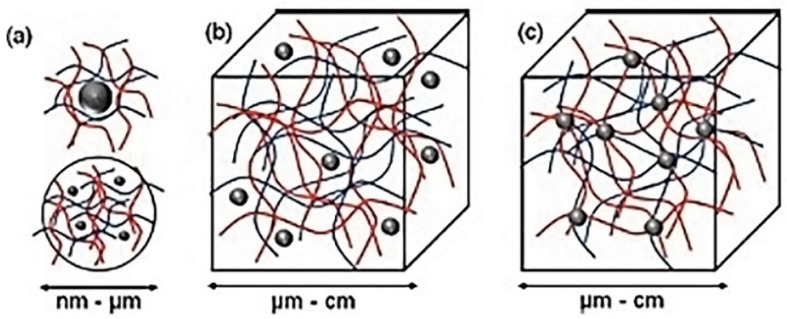
(**a**) The nanoparticle or microparticle stabilizing an inorganic polymer matrix or other polymer nanoparticles. (**b**) Nanoparticles non-covalently dispersed in a hydrogel matrix. (**c**) Nanoparticles which are covalently attached in a hydrogel matrix [40]. https://creativecommons.org/licenses/by/4.0/.

Hydrogels exhibit properties such as swelling and shrinking over several orders of magnitude depending on stimuli exposure. These typically form a cross-linked polymer networks which are highly sensitive to stimuli such as light, solvent composition, solutes, pH, temperature, and electric fields. Significant changes in mechanical properties and thermal response was observed in poly *N*-isopropyl amide hydrogels with the incorporation of gold nanoparticles dispersed in the hydrogel. Typical approaches for producing the nanoparticles embedded in hydrogel is to use preformed nanoparticles and adding it to a hydrogel monomer forming solution. The nanoparticle composition may vary depending on the desired properties. It is important to keep the crosslink density of the polymer matrix high to prevent nanoparticle leaching. This process results in three different types of nanoparticle embedded hydrogels as illustrated in Figure 1 [40].

**Figure 2:**
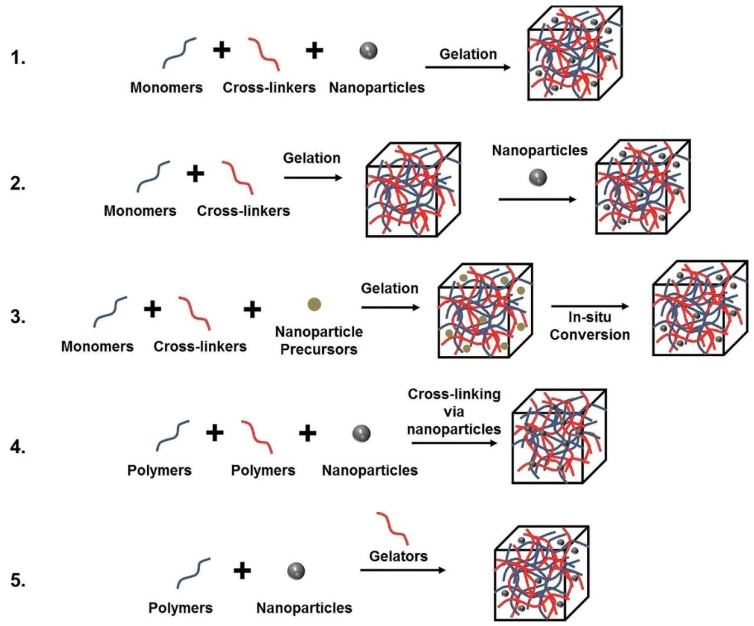
Five main approaches used to obtain hydrogel-nanoparticle conjugates with uniform distribution: (**1**) hydrogel formation in a nanoparticle suspension, (**2**) physically embedding the nanoparticles into hydrogel matrix after gelation, (**3**) reactive nanoparticle formation within a preformed gel, (**4**) cross-linking using nanoparticles to form hydrogels, (**5**) gel formation using nanoparticles, polymers, and distinct gelator molecules. [40]. https://creativecommons.org/licenses/by/4.0/.

Different approaches for obtaining nanoparticle embedded hydrogels are illustrated in Figure 2.

Gold nanoparticles greatly influence the thermal conductivity of the material whilst also imposing antibacterial properties. This had proven to influence the swelling and shrinking of the hydrogel significantly. It was noted that care should be taken when incorporating thermally conductive nanoparticles into hydrogels since, in addition to improving the rate of phase change, the temperature at which a phase change would occur could be affected. The cost of using gold for certain applications generally makes it unfeasible; however, similar results were obtained using silver nanoparticles which are more cost effective. Cobalt and copper nanoparticles could produce a hydrogel which is magnetically sensitive whilst also exhibiting antibacterial properties. Metal oxide nanoparticles exhibit magnetic and conductive properties which could be used in medical applications to mimic muscle movements. The use of non-metallic nanoparticle hydrogels has been associated with catalysis of functional materials such as in the case of using carbon-based nanoparticles to improve drug release whilst providing similar functions compared to metal-based nanoparticles at a lower cost. The combination of using polymeric nanoparticles with characteristics which are different to that of the hydrogel has proven to exhibit properties of both polymeric components [40]. Cyclodextrins exhibit unique properties for encapsulating nanoparticles in their hydrophobic central cavities with great biocompatibility and biodegradability. Cyclodextrins have a stronger a host–guest interface with functional groups when compared to other cavitands [34]. 

### 2.2. Silver/Diatomite Nanoparticles for PCMs

**Figure 3:**
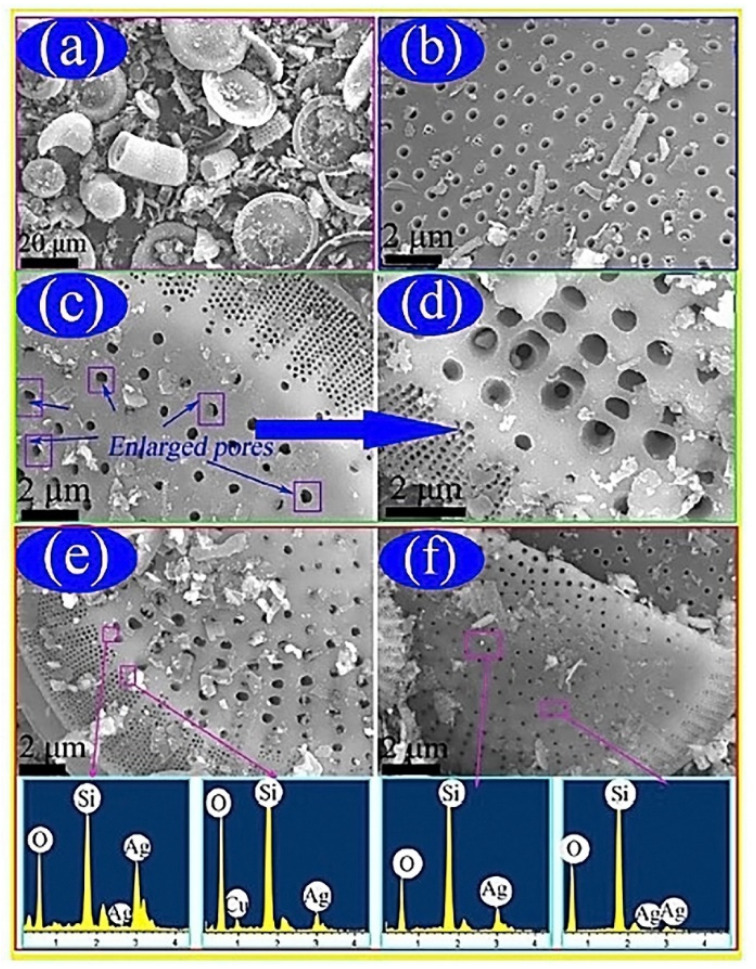
(**a**) Illustrates the disc and cylindrical like structures of the raw diatomite powder. This also indicated impurities blocking the pores of the diatomite. (**b**) After acid treatment, some of the impurities were removed. (**c**) Morphology of the porous structure was maintained after alkali leaching. (**d**) Enlargement of (**c**) indicating where the PEG would disperse the AgNPs in the pores of the purified diatomite. (**e**,**f**) Indicates the deposit of the AgNPs on the surface of the structure. The energy dispersive X-ray spectroscopy spectra confirmed the presence of silicon, oxygen, and silver in the composite, while proper AgNP dispersion was confirmed visually [41].

Silver nanoparticles (AgNP) proved to be a promising additive because of its ability to enhance the thermal conductivity of organic phase change materials. Qian et al. experimented with this property of AgNPs for solar energy applications by using PCMs, since it was found to be the most effective method of latent heat storage. This is due to a higher density of heat that could be stored at a smaller temperature difference between storing and releasing heat energy. Organic PCMs are widely used due to higher latent heat density, suitable phase transition temperature, smaller temperature swings, reasonable cost, and stable physical and chemical properties over long periods of use. Polyethylene glycol was one of the most studied polymers for PCMs, however several problems were associated with handling of the material during phase changes. This was compensated for by packaging technology to produce a shape stabilized PCM. Another approach was encapsulation using inorganic materials for isolation; however, this was associated with high synthesis cost and physical aspects such as chemical stability and flammability. Shape stabilized PCMs adopt good thermal-conductive supporting materials with the ability to maintain a solid shape even when the temperature exceeded the melting temperature of the material. The authors observed that ss-PCMs prepared with diatomite had the greatest energy storing density, thermal conductivity, geological deposits, excellent absorption capacity, low density, chemical inertness, porous structure of 80–90%, and low cost compared to other PCMs used in the building industry. One disadvantage was impurities blocking the pores of the raw materials, but significant improvement was made by treatment of the raw material using the facile alkali-leaching method. The thermal conductivity of the PCM was improved by incorporation of silver nanoparticles due to its high TC-value of 429 W m^−1^ K^−1^ [41]. Diatomite was purified by a purification process using AgNPs. Results for the purification of the diatomite are presented in Figure 3 [41].

**Figure 4:**
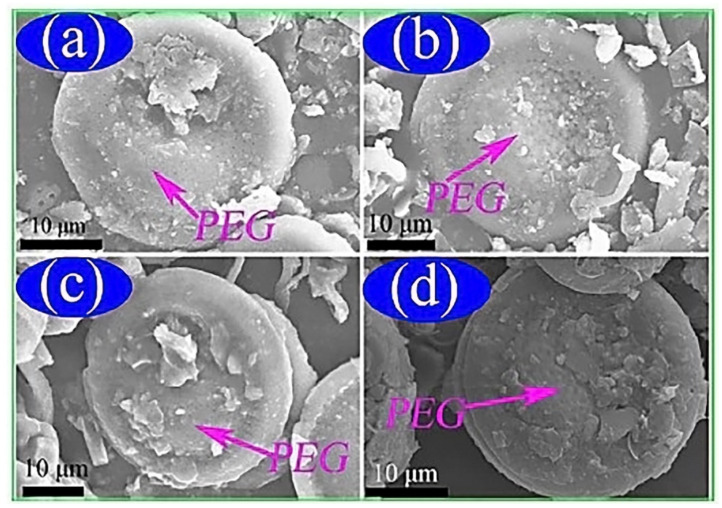
SEM analysis of prepared (**a**) 30%, (**b**) 40%, (**c**) 50%, (**d**) 63% PEG/DtAg ss-PCMs [41].

X-ray diffraction results indicated that the PEG crystal structure was not destroyed after impregnation. A high sample purity was obtained since no deviations from the expected graph pattern was observed. FTIR spectra confirmed the silver in the composite was in pure form and that there was no chemical interaction between the PEG and DtAg. Figure 4 shows that PEG with different mass ratios (30%, 40%, 50%, and 63%) was well dispersed in the porous structure of the DtAg. The multi-porous structure of the diatomite prevented liquids and PEG leakage because of capillary and surface tension forces. As indicated in Figure 4d, PEG was not leaked from the surface of the composite when the PEG was in the melting state because the hot spot created by the electric beam made the investigated PCM melt locally when conducting SEM. From this, the maximum appropriate mass ratio of the PEG in the composites was determined to be 63% [41].

Thermal behavior of the samples was measured using DSC and the data obtained for the respective samples are presented in Table 2.

Thermal conductivity was measured using a hot disk thermal constant analyzer at room temperature. Measurements indicated an improvement of 127% for the composite PCM compared to raw diatomite. This was due to the improved thermal conductivity if the purified diatomite as well as the high thermal conductivity of the silver. XRD, FTIR, TGA, and a 200-cycle test results proved excellent chemical compatibility and the improved supercooling extent, stability, and reliability of the created PEG/DtAg ss-PCMs [41].

### 2.3. Thermal Interference Composites (TICs) Using Carbon Nanotubes

Thermal interference composites (TICs) contain both non-electrically conductive micron sized fillers and electrically conductive nanoparticles which are blended with a polymer matrix. This blend increases the bulk thermal conductivity of the polymer composite materials whilst decreasing the thermal interfacial resistances between the matrix material and the nanoparticle surface it is adhered to. Materials of this composition exhibit no electrical conductivity whilst the incorporation of nanoparticles promoted less phase separation of micron sized filler particles. This provided an electrically insulative material whilst facilitating heat transfer from the source. It was noted that, when applying the material to a heat source, no air gaps were present at the interface as this would inhibit the electrically insulative material from effectively removing the heat from the source. The invention stated an organic polymer matrix comprised of either polydimethylsiloxane resins with any functional chemical elements, epoxy resins, acrylate resins, polyimide resins, fluorocarbon resins, benzocyclobutane resins fluorocarbon resins, fluorinated polyallelic ethers, polyamide resins, polyimidoamide resins, phenol resol resins, aromatic polyester resins, polypropylene ether resins, bismaleimide triazine resins, fluororesins, and any other resins which are electrically non-conductive. These can be used in curable, gel, grease, or phase change material form which would hold the nanoparticle components together. The micron sized fillers are typically thermally conductive materials which could reinforce the polymer matrix or not. Loading density is between 10% and 95% of the final weight of the produced material. Filler particles range in size from 1–100 microns although between 10 and 50 microns are preferred. These include fillers such as amorphous-, fumed or fused silica, quartz powder, carbon black, graphite, diamond, aluminum hydrates, metal nitrides, metal oxides, or any combinations of these. Electrically conductive nanoparticles are typically made from gold, aluminum, platinum, palladium, graphite, silver or copper. Semi conductive materials such as doped silicon or silicon carbide could also be used. These range in size between 1 to 250 nanometres, however between 10 and 100 nanometers are preferred. To facilitate nanoparticle and filler binding to the organic polymer matrix a suitable solvent such as isopropanol, 1-methoxy-2-propanol, 1-methoxy-2-propyl acetate, toluene, xylene, *n*-methyl pyrrolidone, dichlorobenzene, or any combination could be used. Binding is typically performed at temperatures ranging between 20 °C and 140 °C and under a vacuum of 0.5 Torr–250 Torr to remove any volatiles and excess solvent. This can be combined with a curing catalyst of about 10 ppm in the melt composition and less than 2% weight of the total curable composition. Examples of these catalysts include bisaryliodonium salts, radial curing catalysts or group 8–10 transition metals in the case of silicone resins. These may differ depending on the type of resin used. Nanoparticle containing TICs can have a thermal conductivity of <3 times that of TICs which do not contain any nanoparticles. This is due to the creation of thermally conductive pathways between the micron sized filler particles which are enhanced by smaller pathways created by the conductive nanoparticles which typically comprises between 3–50% of the overall composite weight. Loading the polymer matrix with semi conductive nanoparticles more than 50% weight might lead to TICs which are able to conduct electricity effectively. High nanoparticle loads increase the viscosity of the polymer solution which makes handling difficult. Curing occurred by using UV light, microwave, electron beam or combinations thereof at temperatures ranging between 20 °C and 250 °C for 90 s to 60 min. Curing pressure ranged from 1 atmosphere up to 5 tons per square inch, however typically this only goes up to 100 pounds per square inch for practical reasons. [42]. By incorporating 4% of total weight carbon nanotubes in a polypropylene matrix for a TIC exhibited a higher thermal conductivity compared to copper, synthetic diamond, and boron nitride nanoparticles. It was also found that mechanical properties for the carbon nanotube incorporated composite was greater compared to the other three nanoparticle types [43].

### 2.4. Mesoporous Silica MPSiO_2_ Nanoparticles

**Figure 5:**
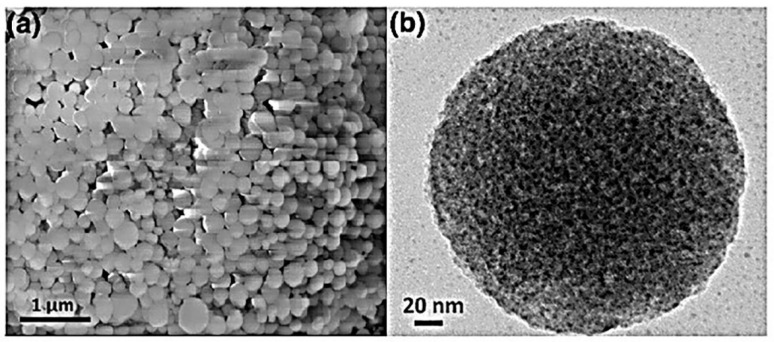
(**a**) SEM and (**b**) TEM micrographs of MPSiO2 particles [2].

Authors Motahar et al., used mesoporous silica nanoparticles (MPSiO_2_) given its porous and morphological characteristics to investigate the thermal conductivity of a PCM which contains the nanoparticles. The MPSiO_2_ was dispersed in *n*-octadecane and the rheological behavior was examined. *N*-octadecane required degassing due to its high dissolved air content followed by the addition of nanoparticles and melting under vacuum. The PCM was stirred using a mechanical stirrer at 1000 rpm and 50 °C followed by sonication to ensure uniform dispersion of nanoparticles. Distilled water and a mixture with ethylene glycol (50%) was used as reference standards with TC and viscosity values of octadecane obtained from literature. Investigation of samples was performed using SEM and TEM to present micrographs as shown in the figures. MPSiO_2_ exhibited spherical morphology as presented in Figure 5 with an average particle size of 350 ± 100 nm and pore size of around 5.0 nm [2].

**Figure 6:**
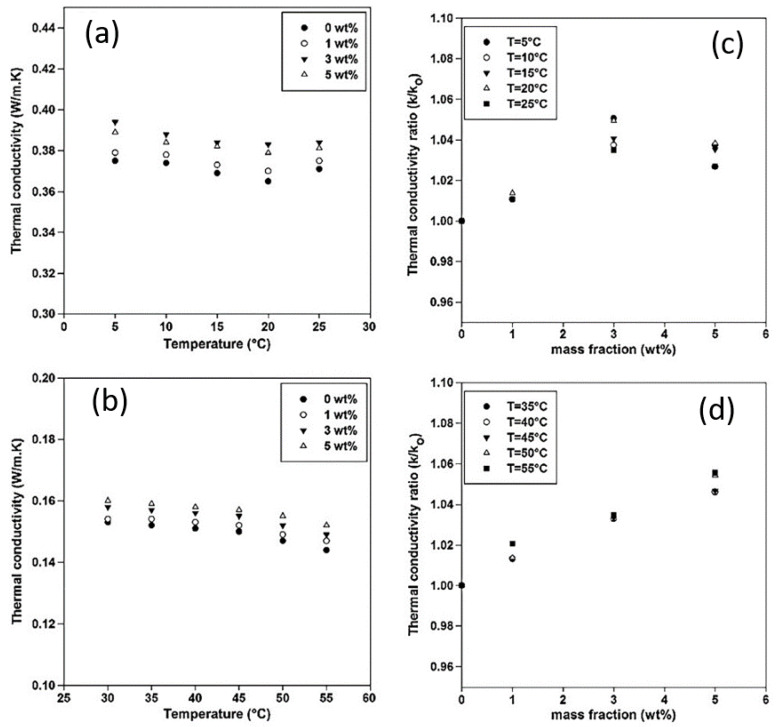
Left: Thermal conductivity of PCM/MPSiO_2_ as a function of temperature for carious mass fractions of nanoparticles—(**a**) solid phase, (**b**) liquid phase. Right: Thermal conductivity ratio with respect to nanoparticle concentration at different temperatures—(**c**) solid phase, (**d**) liquid phase [2].

Experiments were performed at different mass fractions of MPSiO_2_ and temperature as indicated in the graphs of Figure 6.

As seen from the graphs in Figure 6 above the TC decreased with an increase in temperature. This occurred until the crystalline regions became unstable when the melting point was reached which resulted in a TC increase. This confirmed that for a morphologically stable PCM the TC decreases with increasing temperature and increases with further loading of nanoparticles. Rheological property investigation found that the PCM/MPSiO_2_ behaved like a Newtonian fluid at all temperatures for φ_m_ ≤ 0.01, however for higher loads of nanoparticles non-Newtonian behavior was observed. Viscosity increased when the PCM was in a melted state. Thus, for 5% weight MPSiO_2_/PCM, an increase in viscosity was observed at 35°C which corresponded to the melting point of the sample [2].

### 2.5. Disperse Alumina Nanoparticles (Al_2_O_3_)

Ho and Gao based created a nanoparticle-embedded PCM which was prepared by an emulsion technique using a non-ionic surfactant to disperse alumina (Al_2_O_3_) nanoparticles in paraffin (*n*-octadecane). The materials effective thermophysical properties—including latent heat of fusion, density, dynamic viscosity, and thermal conductivity—were investigated. Alumina nanoparticles were first coated with the non-ionic surfactant by a third of its mass fraction before dispersing the nanoparticles in paraffin using an ultrasonic disruptor for 3 h until full dispersion occurred. A constant temperature bath was used with temperature set above the melting point of the liquid paraffin. This technique was used to produce two samples of 5.0 wt % and 10.0 wt % alumina nanoparticles. The samples were then analyzed using DSC measurement at a heating rate of 2 K/min and a temperature range of 20–40 °C [44]. Viscosity increased with nanoparticle loading density as expected. At a temperature of 30 °C, relative increases of nearly 20% and more than 28% in the dynamic viscosity were found for 5 wt % and 10 wt % alumina nanoparticles samples respectively. This appeared about 10–4 times greater than the relative enhancement in the thermal conductivity if compared at the same nanoparticle mass fractions respectively. It was therefore noted that with an increase in temperature as the viscosity of the solution decreases the thermal conductivity would increase correspondingly. Since the increase in viscosity was of greater magnitude than that of the increase of thermal conductivity for the respective samples reasonable doubt emerged given the efficacy for natural-convention-dominated thermal energy storage applications of the experiment [44].

Statements made in the article regarding the effects of nanoparticle concentration on the viscosity of a nanofluid was supported by Kang, Kim, and Oh where a mathematical approach was taken using Einstein’s relation to relate the effective volume fraction to the thermal conductivity of a nanoparticle solution. In the experiment conducted the thermal conductivity was predicted using this method for ultra-dispersed diamond (UDD) powder in ethylene glycol, silver, and silica in water respectively. It was found that the thermal conductivity of the ethylene glycol solution was increased by up to 70% for a 1% UDD concentration with similar increases also noted for the silver and silica in water. The exact thermal conductivity could have not be measured accurately due to the temperature uncertainty using the transient hot wire method [45]. 

### 2.6. Microencapsulated PCM Nanoparticles

Salaün et al. prepared four nanoparticle PCM microcapsules and investigated their thermomechanical properties. Sample ARD was prepared by microencapsulation of a ternary mixture of *n*-hexadecane, *n*-eicosane, and tetraethyl orthosilicate in a 48/48/4 wt % ratio. The in-situ polymerization occurred under constant stirring. Prior to encapsulation, all three polymerization reactants were emulsified into an aqueous solution of Arkofix NM containing a binary mixture of Tween^®^ 20 and Brij^®^ 35 at pH 4 at 8000 rpm using ultra turrax high speed homogenizer. Following this, the reaction was heated to 60 °C with stirring at 400 rpm for 4 h until the end of the polycondensation reaction. The pH of the solution was increased to 9 with 50 wt % triethanolamine solution. The suspension was cooled to 25 °C and filtered, followed by washing of the microcapsules twice with methanol and distilled water. Self-condensation of amino resin around the core material droplet occurred due to high surface activity which enriches the resin molecules within the interface. Hydrophilic/hydrophobic interactions of the partial methylolated melamine enhanced the concentration of the reactive resin molecules in the boundary layer. For this reason, the resin condensation proceeded at a much faster pace in the boundary layer than in the volume phase, which resulted in the formation of tougher microcapsule capsule walls [46].

Sample 18 was prepared by 4 g of DSP and 2 g of distilled water was mixed and added to a solution of 0.5 g of mixture of non-ionic surfactants of 1/3 of Span^®^ 85 and 2/3 PEG 400 dioleate in 7 g of *n*-alkane as for the ARD sample to obtain the first solution. The solution was then stirred for 15 min at 9500 rpm whilst performing a reduction in droplet size by homogenizing the emulsion during stirring. Similarly, emulsion 2 was prepared by homogenizing 8 g of 5% PVA solution in 8 g of *n*-alkane. The particles in sample 18 were prepared by shearing under high speed for the two prepared emulsions with 3 g of MDI to crosslink the shell at 50 °C for 30 min. The formed polymer nanoparticles were observed using SEM [46].

Preparation of samples E2 and H/E used the resultant solutions of sample 18 containing the nanoparticles in a paraffinic medium. This was emulsified in an aqueous solution containing 4 g of Tween^®^ 20 in 100 g of distilled water and 9.2 g of Arkofix NM. 30 wt %. A citric acid solution was used to reduce the pH to 3 whilst stirring at 8000 rpm under room temperature with a homogenizer. After 3 min, the reaction mixture was heated to 60 °C, and stirring continued at 400 rpm for 4 h using a blade stirrer until the polycondensation reaction was complete. The microparticles were recovered by filtration and washed with methanol and distilled water followed by drying at room temperature overnight. From the two paraffinic solutions obtained in the preparation of sample 18, two types of multinuclear microparticles was obtained ‘E2′ and ‘H/E’ respectively [46].

For the ARD sample the shell size ranged between 1–3 µm. For sample 18 polymer nanoparticles were produced with diameters of about 50 nm. Aggregation occurred due to high particle density. SEM analysis of sample E2 proved the high nanoparticle density and configuration of polymer nanoparticles inside the shell. The shells included 68% paraffin allowing the polymer nanoparticles to generate a very dense and thermally insulating structure. Latent heat was determined to be equal to 176 J/g. It was found that the temperature at which the solid–liquid transition started was the same, but the temperature span was 50% larger for the nanoparticles based PCM compared to the conventional microcapsules. This modification in the extended temperature span of the phase change is beneficial for textile application because a specific temperature would be maintained under a broader heat flux amplitude [46].

The ARD sample had a higher thermal conductivity than sample 18. Increases in temperature resulted in both samples become more thermally resistive. Since samples E2 and H/E were 60–70% more insulating than paraffin [46].

Incorporating PANI into a matrix can greatly improve the energy storage capacity of a PCM. It was found that by microencapsulation of paraffin wax with PANI exhibited an energy release capacity of between 22–121 J/g respective to the ratios of PANI/paraffin wax used [47].

## 3. Nanoparticles for Improving Electrical Conductivity

Conductive electrodes and electric circuits that remain stable under repetitive mechanical forces are highly desirable and typically used in the new era of flexible display technology, energy related devices, smart clothing, and field effect transistors. Flexibility, electrical conductivity, and strength are mutually exclusive parameters in designing this type of material. Commonly insulation techniques are used where the conductive material is covered with a flexible elastomer such as in the case of electric wiring. New techniques are being developed to incorporate the conductive material inside the reinforcing structure which allows the typically non-conductive material to conduct an electrical current. For this purpose, an article by Park et al. investigated the use of an SBS rubber fiber material impregnated with silver nanoparticles. The resulting material was able to maintain effective bulk conductivity whilst overcoming large deformations. Applications of these include stretchable antennas or wearable electronics [48].

### 3.1. Increasing the Seebeck Coefficient with Conductive Nanoparticles

The conversion of thermal energy into electrical energy is known as thermoelectric conversion. This can result in the generation of electricity or electronic refrigeration. When a temperature gradient is applied to a thermoelectric couple, this allows for the transportation of electrons (n-type) and is hole-transporting (p-type). Electrons diffuse from the warm end of the thermoelectric couple and will collect at the colder end which produces an electrostatic potential. The ability of a material to perform this is called the Seebeck effect [49]. The net Seebeck coefficient of a material is directly related to the strength of the electric field inside the material and is measured as voltage per unit temperature difference [50].

The opposite effect occurs when a voltage is applied to the thermocouple as the electrons will attempt to arrange themselves in a similar order as before the current was applied. This results in an absorption of energy at the side where current is applied and releasing the energy at the other end. This is also known as the Peltier effect. Advantages of this method is the absence of any moving parts as traditionally expected with electricity generation or refrigeration. Efficiency of thermoelectric materials are strongly associated with the dimensionless ZT-value of the material. Thermodynamic efficiency is measured as *ZT* = (*α*^2^*σ*/*κ*)T where *α* relates to the Seebeck coefficient of the material, electrical resistivity σ, the thermal conductivity κ and the absolute temperature T [15]. For high performance thermoelectric materials, the efficiency is also dependent on a material’s ability to have a high electrical conductivity, a large Seebeck coefficient, and low thermal conductivity. This would result in a larger temperature difference between the ends at which stress is applied and released. This stress is related to the application of a temperature difference or electrical current. By enhancing the *ZT*-value of a composite the system can rely less on thermal and electrical transport mechanisms which forms the basis for a nanostructural approach to enhancing the thermoelectric performance of a material. Single crystals of nanoparticles exhibit the best electrical conductivity due to their absence of grain boundaries scattering charge carriers; however, ZT-value optimization can only be achieved by elemental doping to adjust the carrier concentration. When the size of the thermoelectric material is reduced, especially to the nanoscale system the mechanism of operation approaches a scale which can be compared to that of electron behavior in any direction. This increases the Seebeck coefficient of the material whilst reducing the thermal conductivity due to increased phonon scattering. For this reason, thermoelectrical materials on nanoscale typically exhibit thermal conductivity values much lower than that of the bulk material as in the cases reported for Bi_2_Te_3_ and silicon nanowire between 10–20 nm. Thermal conductivity could also be reduced by the incorporation of more grain boundaries however, this would decrease the carrier mobility which reduces the efficacy of the material [49,51].

#### 3.1.1. Bi_2_Te_3_ Nanoparticle Alloys

Li et al. investigated the thermoelectric performance of a high-performance thermoelectric material using Bi_2_Te_3_ alloys. It was found that mechanical milling of the bulk material into a nanopowder resulted in an increase in the ZT-value of Bi_2_Sb_2−x_Te_3_ to 1.4 at 373 K. This was attributed to a decrease in thermal conductivity by larger scattering at the grain boundary and the presence of nanoparticles. Also investigated was the development of a melt–quench–anneal–spark plasma sintering method to form bulk nanostructural p-type Bi_0.52_ Sb_1.48_Te_3_ with a ZT-value of 1.56 at 300 K. Also obtained was a maximum ZT = 1.47 at 438 K for Bi_2_Te_3_/Sb_2_Te_3_ bulk nanocomposites with laminated nanostructures using hydrothermal synthesis and hot pressing. A 20% increase in the ZT-value was obtained by the incorporation of SiC nanoparticles into the Bi_2_Te_3_ alloy at 0.2 volume percent. Further addition of SiC nanoparticles resulted in a decrease of the ZT value since the SiC particles had a higher electrical resistivity and thermal conductivity than that of the alloy. An increase in microhardness and fracture toughness was observed which improved the ability to handle and manufacture the composite material [49].

#### 3.1.2. CoSb_3_ Nanoparticles

CoSb_3_ based skutterudites were also investigated due to the compound’s high effective mass and carrier mobility. It was found that the CoSb_3_ nanoparticles had a very high thermal conductivity of 10 Wm^−1^ K^−1^ compared to that of Bi_2_Te_3_ (1.0–1.5 Wm^−1^ K^−1^) which resulted in a lower ZT-value. This was overcome by incorporating a smaller filler elemental ion, such as iron, inside the cage-like structure formed at the center of the parent compound which was able to reduce the thermal conductivity to 1.4 Wm^−1^ K^−1^. This was due to the inserted ion rattling at the equilibrium position which generated a significant number of phonons. It was found that the filler elemental ion with the largest cage filing fraction resulted in the largest increase in ZT-values. Only filler elements with electronegativities larger than 0.8 were able to enter the cage-like structure of the central metal atom in the compound. It was also stated that a good thermoelectric device requires both n- and p-type legs to overcome possible failure due to thermal stresses. This concluded that CoSb_3_ was excellent choice for medium temperature applications due to the incorporation of both components [49].

#### 3.1.3. Ag_1−x_Pb_18_SbTe_20_ Nanoparticles

Ag_1−x_Pb_18_SbTe_20_ was successfully synthesized and possessed a NaCl symmetry structure with nanoparticles scattered in the PbTe matrix. This proved to have a very high ZT-value of 2.2 at 800 K making this an excellent thermoelectrical material. The study found that the thermoelectrical materials were very sensitive to chemical composition especially regarding lead content. Use of an annealing treatment to the thermoelectrical material resulted in great success. After treatment, the thermal conductivity of the material was reduced whilst simultaneously increasing the electrical conductivity. This was attributed to the formation of a nanostructure which favored electron transport and phonon scattering which resulted in a significant improvement of the ZT-value to 1.7 at 703 K. The compound was resynthesized to provide a low thermal conductivity value of 0.3 Wm^−1^ K^−1^ and ZT-value of 1.59 at 673 K. Comparable values were obtained by substituting silver for potassium and sodium with slight deviations in ZT-values and temperature [49].

Half-Heusler compounds prove to be more environmentally friendly compared to compounds containing lead. Compound symmetry was interpreted as two interpenetrating cubic face-cantered-cubic motifs with an embedded cubic motif at the center of the structure. The chemical structure of these compounds are typically MgAgAs type systems. Half-Heusler compounds typically have high thermal conductivity values resulting a lower ZT-value compared to previously mentioned compounds [49].

#### 3.1.4. Metal Oxide Nanoparticles

Oxide materials usually exhibits a high degree of thermal stability and oxidation resistance which makes them excellent for use in high temperature applications. Previously, these materials had been ignored due to their low electrical conductivity but have gained new interest with recent discovery of NaCo_2_O_4_. NaCo_2_O_4_ possesses a similar structure to the superconductor YBaCuO sandwiched between a CoO_2_ and sodium layer which forms laminated sheets on the outside surfaces. NaCo_2_O_4_ has an unusually large Seebeck coefficient because of a strong electronic correlation effect in the material. Coupled with a high electrical conductivity the power factor of the thermoelectric material is 5000 Wm^−1^ K^−1^ which is higher than that of previously mentioned Bi_2_Te_3_ (4000 Wm^−1^ K^−1^) [49].

Typically, SiO_2_ nanoparticles exhibit poor electrical conductivity and microwave absorptive properties. When heterogeneous nitrogen, carbon, and chlorine atoms were incorporated on the surface these nanoparticles proved to be effective electrical conductors with the ability to absorb microwaves because of dielectric loss due to improved electrical conductivity. This makes the particles suitable for applications such as wireless communications and anti-radar materials, even when incorporated into a suitable noninterfering matrix [52].

### 3.2. Silver Nanoparticles

Silver nanoparticles are widely known for their excellent thermal and electrical conductivity. Dermanaki Farahani et al. focused on the electrical conductivity of these nanoparticles for incorporation into a polymer matrix and used two different coating methods onto a carbon fiber substrate to incorporate electrical conductivity whilst also investigating the efficiency of the application technique. The two techniques were application by spraying the nanoparticle polymer solution or by casting. The applied nanoparticle polymer was used as a binder to which a second polymer was applied. This resulted in the fabrication of a ternary biphasic nanocomposite which further improved mechanical resistance properties of the carbon fiber substrate. Polymer-nanoparticle application was performed using an annealing process. The produced materials posed potential aerospace applications such as shielding from electromagnetic interference and lighting strike protection [53].

A binary nanocomposite which consisted of a blend of conductive silver nanoparticle inks and PEDOT:PSS as a conductive binder. For 50 wt % of ink, the desired amount of the mixed conductive ink was dissolved in deionized water with sonication. The solution was then added to a specific amount (1:1 ink to PEDOT:PSS) of PEDOT:PSS in a glass bottle and ultrasonicated. This was followed by stirring at 400 RPM for 3 h. The ternary nanocomposite was an 80:20 blend of epoxy emulsion with PEDOT:PSS and the conductive silver nanoparticle inks. The epoxy emulsion formed was added to the mixture of PEDOT:PSS and the silver inks for improvement of the mechanical resistance of the final coatings. The final nanocomposite contained 50% of the ink which was first solubilized in 10 mL distilled water with ultrasonication. The solution was then added to a weighted 80:20 amount of the epoxy emulsion and PEDOT:PSS. This was followed by stirring at 80 °C for 3 h at 400 rpm. These inks were applied to aerospace grade carbon fiber sheets [53].

Two coating application methods were used namely spraying and casting for each of the conductive ink samples. After application the coated samples were dried at 90 °C for 20 min at 0.15 bar pressure. For both application methods thermal annealing of the coating materials were performed at 140, 180, and 200 °C [53].

The coating density was determined to be 4.2 g cm^−3^ and 4.6 g cm^−3^ for the binary and ternary samples respectively which was lower than the density of the silver inks used. Density of the silver inks were determined to be 7–8 g cm^−3^. The densities obtained are lower than that of copper foils which are currently used in the aerospace industry. It was hypothesized that with increased annealing temperature the coatings would have attained a connecting feature and resulting in a smoother surface above 140 °C. It was thus hypothesized by the authors that the higher annealing temperature would have resulted in densification of the applied ink; however, measurement results were within the margin of error. Impurities were observed in ink samples as sintering occurred during the annealing process. For adequate removal of impurities an annealing temperature above 250 °C was required to improve the electrical conductivity; however, cracking in the material was observed at temperatures above 220 °C [53].

Slightly higher values for the hardness and modulus were observed for the ternary system versus the binary system which was attributed to remaining epoxy in the samples. High binding effectiveness between the nanoparticle polymer and substrate was obtained by using the spray technique. This technique proved to be the preferred method of application. It was noted that, with increasing annealing temperature, nanoparticle polymer binding did become more effective for the casting method; however, only when annealed at 180 °C and above compared to the spray method which showed good binding results from 140 °C [53].

Electrical conductivity of the samples improved with annealing temperature; however, the binary samples indicated the highest electrical conductivity as lowest resistivity. This proved that a higher annealing temperature is preferred to increase the electrical conductivity of the nanoparticle polymer which was determined to be 1.8 ± 0.3 × 10^3^ Ω g cm^−2^ at 140 °C and increased to 4.2 ± 0.3 × 10^−3^ Ω g cm^−2^ when annealed at 200 °C [53].

Applications using 3D printing of PEGDA:PEDOT was investigated by Scordo et al. It was determined that incorporating 5% DMSO as surfactant with the resin resulted in a lower viscosity compared to Triton-X 100 and DMF, respectively. PEGDA:PEDOT with 5% DMSO yielded an electrical conductivity of 0.05 S cm^−1^ [35].

Fratoddi et al. covalently liked silver nanoparticles with a bifunctional thiol ligand 9,9-didodecyl-2,7-bis-trifluoroene which formed a covalently linked network in an organic solvent matrix. The addition of the ligand to form a covalent network provided the composite film with photoconductive properties. The functionalized silver nanoparticles had an electrical resistivity of 4.9 ± 0.3 MΩ and a conductivity of 0.031 mS/m. With the same voltage and light illumination, the electrical resistivity decreased to 3.3 ± 0.2 MΩ with a conductivity of 0.045 mS/m [30].

**Figure 7:**
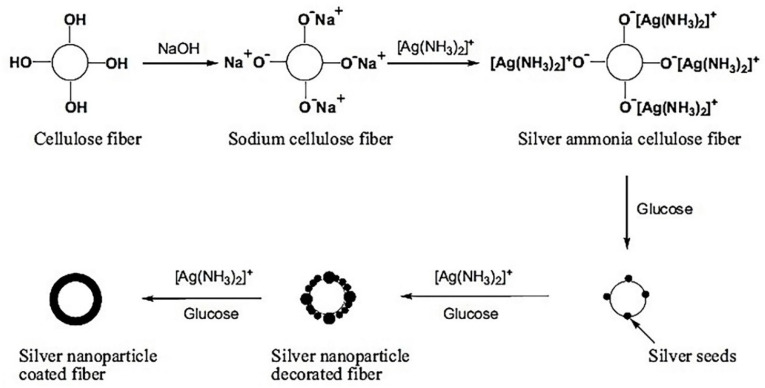
Formation of AgNP coated fibers [54].

Xue et al. coated cellulose nanofibers with silver nanoparticles to incorporate an antimicrobial effect onto the textile whilst also modifying the nanoparticles to promote hydrophobicity. The process of application of the nanoparticles to the fibers is presented in Figure 7. Silver nitrate was used to form the nanoparticles [54].

According to the scheme above, the cellulose textiles were treated with 10% aqueous NaOH solution at room temperature for 10 min followed by rinsing with distilled water. 28% Aqua ammonia was added dropwise to a 0.5 M silver nitrate solution until the solution turned colorless with the formation of [Ag(NH_3_)_2_]^+^. The alkali treated cellulose textile was added to the colorless solution and allowed to react for 1 h followed by transfer to a 0.1 M glucose stock solution for 5 min. The residual [Ag(NH_3_)_2_]^+^ solution was added to the textile in the glucose solution and reaction occurred for 15 min. After reaction, the textile was rinsed and air dried. The samples were then hydrophobized with 3% HDTMS in ethanol for 1 h at 80 °C and cured at 130 °C for 1 h [54].

Bacterial tests were conducted using the bacterial resistance ring method and the reduction of bacterial growth test. The possibility of electrical conductivity incorporated in the fiber modification was measured as the resistance between two points on the original cellulose sample and the modified sample [54].

The water contact angle was determined to be 157.3° for the 5 µL droplet applied to the textile surface. XRD confirmed the presence of pure silver nanoparticles as no impurities were observed in the diagram. The electrical resistance of the textile was very low at 37.0 ohm compared to the infinite result of the original sample which was unable to conduct electricity. The antimicrobial activity was confirmed to destroy 99.99% of bacterial colonies with an inhibition area of 8.78 mm.

Fabrication of a conductive flexible material described by Park et al. consisted of an electrospun non-woven mat of SBS rubber collected on a surface which was treated silicon wafer. After collection, the nonwoven mat was peeled off the silicon surface. The collected material was then dipped into a silver precursor solution of AgCF_3_COO/ethanol. Both precursor and solvent were absorbed by the fibers. Formation of silver shells on the surface of the fibers occurred which could break under strain. Conductivity was maintained by percolation of the silver nanoparticles inside the fibers, as well as bridges formed by the debris of the broken shells. The silver nanoparticle solution was nozzle printed onto the SBS electro spun mat. Absorption of the silver nanoparticles was promoted by the ion–dipole interaction between the hydroxyl groups of the alcohol and the trifluoroacetate anions. This caused the fibers to swell until saturation occurred at 15% total weight concentration. The saturated mat retained 62% of silver nanoparticle content after chemical reduction. FTIR spectroscopy confirmed the presence of precursor inside the fibers which prevented the fibers from shrinking back to dimensions obtained before spinning [48].

It was noted that under low applied stress the conductivity was barely influenced and returned to normal upon relaxation of the fibers. However, electrical conductivity decreased significantly upon excessive stretching to the yield point of the SBS fibers. This resulted in the material adopting a new conductivity value upon release corresponding to that attained under the maximum stretch. Use of the nozzle printing technique could be used to create a circuit on the material [48].

### 3.3. Conductive Carbon Nanoparticles

Böger et al. incorporated electrically conductive carbon nanoparticles into a non-conductive epoxy matrix to detect possible failures in the non-conductive matrix electronically. This could also be applied to other nonconductive matrices. The application potential of this property incorporated into the end use material could be incredibly useful in industry as it omits costly routine inspections and could produce an immediate warning with the use of an indicator in case of component failure.

**Figure 8:**
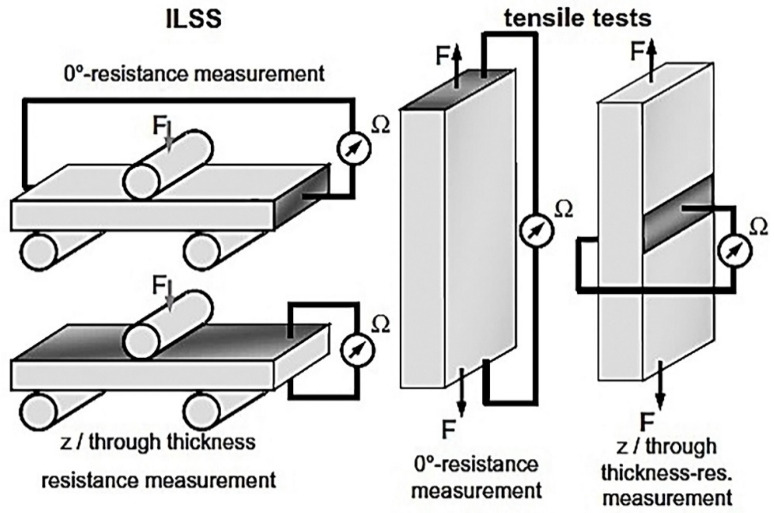
Resistance and tensile test measurement setup [55].

Materials used by the authors included epoxy resin, anhydride hardener, imidazole accelerator and the addition of 0.3% of conductive carbon nanoparticles. Two forms of carbon nanoparticles were investigated namely conductive carbon black and multi walled carbon nanotubes. The modified epoxy system was used to manufacture glass-fiber laminates. Nanoparticles were dispersed in the resin by mixing without the addition of hardener or accelerator and passed through a three-roller mill. The collected resin was bottled and stored at a low temperature to prevent the agglomeration of the carbon nanoparticles. The epoxy/carbon suspension was then mixed with hardener and accelerator using a laboratory vacuum stirrer. Two glass fiber non-crimp fabrics were used on both sides of the epoxy resin to achieve the desired laminate with a stacking sequence stated as (0°, +45°, 90°, −45°, +45°, 90°, −45°, 0°). Heating of the mold was kept at 45 °C to ensure the viscosity of the epoxy matrix remained low for the injection process. During processing an electrical field with a field strength of 33 V/cm was applied to the formed material in the z direction to orientate the carbon nanoparticles forming a conductive network in the matrix. Curing occurred at 80 °C for 4 h and specimens were cut according to industry specification. Two tabs of two ±45° GFRP, 1 mm thick sheets were placed on the side of the specimens. On the fixture side two tabs of aluminum was used. Post curing occurred for 8 h at 140 °C and a conductive silver paint was used on the top and bottom sections of the sheet in the z-direction for conductivity measurement. The painted sides were polished to avoid possible short circuiting. For electrical resistance measurement two opposite sides of the samples were painted with conductive silver paint. For resistance measurement in the z-direction, the upper and lower surface of the test specimens were coated. For the tensile test specimen only a 15 mm wide electrode was applied to both sides in the center of the test specimens [55]. Both setups are presented in Figure 8.

Mechanical property testing involved the inter laminar shear strength, incremental tensile and the dynamic tensile tests. During mechanical testing the resistance was measured by applying a voltage to the specimens [55].

Carbon-black modified epoxy laminates exhibited a resistance change of approximately 25–45%, 10–15% for the nanotube-modified laminates, and approximately 20–40% for the carbon black modified laminates during the resistance change measured in z-direction. The authors contributed the abrupt increase in electrical resistance during measurement to be attributed to the interruption of electrically conductive pathways in the materials by delamination. The delamination that occurred in the fiber plane orthogonal to the z-direction, could have been detected electrically in both measurement directions (0°- and z-direction). Two reasons explaining this phenomenon is first that the delamination was never a purely in-situ failure, but also propagating in thickness. Secondly, the electrical pathways formed by the nanoparticles were randomly 3D oriented in the laminates. Therefore, a resistivity measurement in the 0°-direction is also affected by a delamination in the fiber plane, since the electrical pathways are partly oriented in z-direction. For measurement in the z-direction the resistance was almost unaffected by the application of sheer forces [55].

The effect of resistance during repeated stress were measured and it was determined that only after the fifth cycle residual strain was observed. Electrical resistance increased with amount of load cycles applied, however this occurred to a magnitude of 0.05% after the first five cycles with an irreversible resistance change of 0.02%. Both reversable and irreversible resistance change values increased to 0.52% and 1.4% respectively after the 11th cycle. Epoxy modified with carbon black nanoparticles exhibited the highest sensitivity of between 10–20% regarding irreversible resistance changes [55].

It was concluded that the carbon black modified matrix was more sensitive the applied stresses than multiwall carbon nanotubes due to the difference in structure. Carbon nanotubes are larger in size, thus nanoparticle contact occurs more frequently with neighboring nanotubes promoting their ability to conduct an electrical current [55].

Dynamic tensile tests performed on the specimens indicated that an increase in electrical resistivity occurred upon delamination with resistance reaching up to 100% when a large part of the material was delaminated. The change in resistance was thus concluded to be related to the crack density in the sample [55].

### 3.4. Electromagnetic Interference (EMI) Materials

#### 3.4.1. Nickel Nanoparticles

Los, Lukomska, and Jeziorska investigated composites with filler materials which would reflect electromagnetic radiation at 1–2 GHz with a split post dielectric resonator. This is a typical range for every day devices used by consumers. Polyether sulfone was the chosen matrix for the investigation with a thickness of 2.8 mm. Results indicated that by incorporating 7.0% nickel filaments into the matrix an EMI shielding effectiveness of 87 dB was obtained. This was followed by nickel fibers 58 dB with reducing effectiveness observed with increasing filler/particle size [30].

#### 3.4.2. Fe_3_O_4_ Nanoparticles

Ferreira, Bayraktar, and Robert investigated changes in electrical conductivity and magnetic properties of an aluminum matrix material using Fe_3_O_4_ nanoparticles. Fe_3_O_4_ powder of 99.62% purity was used as filler material and combined with aluminum powder. A ceramic oven was used to form the composite material at 600 °C. Three samples were produced with Fe_3_O_2_ nanoparticle compositions 10%, 20%, and 30% respectively [56].

It was noted from SEM and optical microscope images that the nanoparticles were dispersed homogeneously around the grain boundaries of the aluminum. This was attributed to rapid mixing as the composite was being heated in the ceramic oven. Density of the Fe_3_O_4_ nanoparticles in the composite varied slightly between 2.5 g cm^3^ and 3.0 g cm^3^ [56].

Electrical conductivity measurements indicated that an increase in Fe_3_O_4_ nanoparticle loading resulted in an increase in electrical resistivity relating to a decrease in electrical conductivity. Magnetic saturation increased with Fe_3_O_4_ nanoparticle composition to 13.43 emu/g for the 30% Fe_3_O_4_ containing sample [56]. Electromagnetic shielding effects could be improved by annealing the composite in an H_2_ environment and increasing the materials thermal conductivity to dissipate the heat absorbed by the electromagnetic radiation [57].

#### 3.4.3. Reduced Graphene Oxide (GO) Nanoparticles

Yang et al. produced an electrically conductive reduced graphene oxide (RGO)/cellulose nanofiber film of 23 µm thickness for effective EM shielding. GO has an ultra-high electrical conductivity of 6000 S cm^−1^ and thermal conductivity of 5000 W m^−1^ K^−1^. It is typically impossible to achieve the theoretical maxima for electrical conductivity of GO since strong van der Walls forces between particles inhibits their homogeneous dispersion on a substrate. The films were obtained by mixing a 3.63 mg g^−1^ aqueous solution of GO and a 3.54 mg g^−1^ aqueous solution of cellulose nanofibers using a magnetic stirrer for 6 h at ambient temperature. This was followed by sonication of the dispersion for 30 min and filtration using a cellulose ester membrane. The obtained films were then dried between two sheets of paper with pressure applied at ambient temperature. Reduction occurred by placing the films in hydroiodic acid for 5 min followed by rinsing with ethanol. The films were then dried between two sheets of paper under pressure at ambient temperature. At 50% RGO/cellulose nanofiber an electrical conductivity of 4057.3 S m^−1^ was obtained and an EM shielding effectiveness of 26.2 dB in the X-band [58].

Fang et al. developed a self-healing GO-PDMS-urea film of 1 mm thickness. Various layers of graphene were dispersed in THF by ultrasonication followed by mixing with PDMS–urea solutions to form composite mixtures. By casting the composite solutions in a Teflon well freestanding composite films with the graphene weight fractions of 3, 5, 7, 10, and 12 wt % were prepared. Following casting the respective samples were placed into an oven at 60 °C for 30 min to cure. Best electrical conductivity results were obtained with the 12 wt % sample which had an electrical conductivity of 81.5 S/m. The 10 wt % composite film was subjected to tensile tests and self-healing was promoted by subjecting the sample to THF vapor for 2 h. After self-healing electrical conductivity recovered to 95% of the original value [59].

## 4. Recovery and Disposal of Nanoparticles in Composites

The environmental impact of the use of polymeric materials and toxicity of materials are becoming more of a concern. Recycling and disposal of nanocomposite materials can be challenging due to their multicomponent nature. Ohde et al. had developed a method of recycling high value metallic nanoparticles where the composite material is solvated and extracted using a water in CO_2_ microemulsion technique. The authors were able to recycle and resynthesize lead nanoparticles effectively up to five times [60].

Tiwary et al. developed a technique to recycle printed circuit boards by using low temperature ball milling to break the composite into nanoscale particles. The procedure was performed using a self-designed cryo-mill which operated at a temperature of 154 K in an inert atmosphere of Argon. Milling of the printed circuit boards was performed using a single steel ball in the milling chamber. Constant temperature was maintained with a continuous flow of liquid nitrogen. It was noted that, for larger composites, milling into nanoscale powders could take between 1–3 h under the same conditions. Milling composite materials into nanopowder increased the separation efficiency by material density significantly compared to other methods which did not involve nanoscale recycling. Fluid separation proved very effective in isolating individual components of the nanopowder according to particle density [37].

Typically, most thermoplastic composites could be ground into a powder and carbonized. The formed carbon nanoparticles could then act as reinforcement for other recycled materials such as polymers which typically experience reduction of mechanical strength upon recycling due to polymer chain breakage [61].

## 5. Conclusions

The properties of nanoparticle containing composite materials are largely affected by the size of nanoparticles used. Incorporation of nanoparticles to increase the thermal or electrical conductivity of a material typically effects the mechanical properties of the composite. Smaller nanoparticles tend to have higher reactivity due to a high surface area to volume ratio, whereas larger particles may act more as reinforcement [5]. Surface contact between neighboring nanoparticles will create pathways through which thermal radiation and electrical current could be conducted. The conductivity through these pathways depends on the thermal and electrical conductivity of the particles used. Electrical conductivity can be improved by functionalizing nanoparticles to create a molecular network inside the matrix with properties depending on the type of functionalization [27]. Hydrogels prove to be very promising for thermal energy storage with their effectiveness greatly improved by nanoparticles. Hydrogels are highly effective PCMs and recent trends have come to produce hydrogel nanoparticles for use in drug delivery [62]. PANI is considered one of the most promising supercapacitor electrode materials because of its high conductivity, easy synthesis, excellent capacity for energy storage, and low cost. When used in a PCM, PANI experiences a sharp degradation in performance due to repeated swelling and shrinkage. However, when combining PANI with carbon nanomaterials this had proven to reinforce the mechanical stability as well as maximizing the energy storage capacitance of the polymer [63].

Non-spherical nanomaterials, such as silver nanowires, proved to contribute higher increases in thermal and electrical conductivity of composites compared to spherical nanoparticles with a percolation of less than 1% volume fraction due to increased particle contact [64].

SiO_2_ nanoparticles are non-conductive and are associated with low microwave absorption; however—with surface modification of nitrogen, carbon, and chlorine atoms—these proved to be good conductors with excellent microwave absorptive properties [52]. When applying a nanoparticle polymer solution onto a substrate the spraying method proved to be more effective. Higher annealing temperatures not only improved binding of the applied nanoparticle polymer, but also increased the electrical conductivity substantially [53].

The use of nickel nanoparticles in a polyether sulfone matrix exhibited the highest electromagnetic shielding effectiveness. As particle size increased, EM shielding effectiveness decreased significantly [30]. Electromagnetic shielding can be improved by influencing the annealing atmosphere and enabling the material to dissipate the absorbed heat from electromagnetic radiation [57]. For conductivity enhancement of a matrix the first choice tends to be the use of metallic nanoparticles; however, these tend to be much heavier than carbon alternatives [53]. Improvements have been made in this regard by using carbon nanostructures and nanoparticles to achieve similar electrical and thermal conductivity to metallic nanoparticles such as copper [65].

A typical problem when using nanoparticles in a polymer matrix is the occurrence of agglomeration due to high particle surface energy as well as potential attractive forces. Ultrasonication is still the preferred method of dispersion since it is a fast and effective process with parameters which could be easily controlled [66]. Ultrasonication at low frequency can be relatively undamaging to nanoparticles, whilst at high frequency it has the potential to break C-C bonds such as in the case of the dispersion of nanoclays [67].

Graphene oxide nanoparticles exhibit ultra-high electrical and thermal conductivity, however only with more recent developments have materials been developed with electrical and thermal conductivities closer to theoretical values [58]. Electrically conductive composites typically exhibit loss of conductivity under strain as a result of interruption in the conductive pathways. Recent studies have delivered promising results for self-healing materials to overcome this issue.

Future trends are leaning towards the development of stretchable conductors. Wang et al. developed a styrene ethylene butylene styrene (SEBS) PEDOT:PSS doped composite film. The material maintained an electrical conductivity of 4100 S/cm under 100% strain reducing to 100 S/cm under 600% strain which was the highest among stretchable conductors. The electrical conductivity reduced to 3600 S/cm after 1000 cycles to 100% strain [68].

Recyclability of composites are becoming increasingly important as composite waste projections indicated a sharp increase. Current waste disposal techniques, such as incineration releases hazardous gasses into the atmosphere when incinerating polymeric materials. A 100% effective material recycling rate is difficult to attain; however, by using methods such as low temperature ball milling it has become possible. This would have a significantly positive impact on composite recycling and reducing their polluting impact on the environment [37].

## Figures and Tables

**Table 1:** Effect of nanoparticle size on matrix properties [5] (http://creativecommons.org/licenses/by/4.0/)

Properties	Feature Sizes (nm) at Which Changes Might Be Expected
Catalytic activity	<5
Making hard magnetic materials soft	<20
Producing refractive index changes	<50
Producing super paramagnetism and others electromagnetic phenomena	<100
Producing strengthening and toughening	<100
Modifying hardness and plasticity	<100

**Table 2:** DSC results of different PEG/DtAg ss-PCM samples [41]

Samples	PEG Mass Ratio (wt %)	Melting Process		Solidifying Process	
		H_M_ (J/g)	T_M_ (°C)	H_S_ (J/g)	T_S_ (°C)
PEG PCM	100	180.3	60.51	164.6	38.5
ss-PCM1	30	51.5	59.21	46.1	38.63
ss-PCM2	40	70.2	58.86	64.1	39.21
ss-PCM3	50	88.4	59.13	80.3	40.54
ss-PCM4	63	111.3	59.45	102.4	41.02
ss-PCM5	63	110.7	59.83	103.3	39.54
